# Factors affecting intention to adopt Islamic financing: Evidence from Morocco

**DOI:** 10.1016/j.mex.2021.101523

**Published:** 2021-09-21

**Authors:** Omar Boubker, Khadija Douayri, Abdelaziz Ouajdouni

**Affiliations:** a Higher School of Technology, Ibn Zohr University, Laayoune, Morocco; b Research Laboratory in Business Management (LaRGE), Research Team in Development and Project Management (EDPM), National School of Business & Management, Ibn Zohr University, Agadir, Morocco; c Higher School of Technology, Mohammed First University Oujda, Morocco; d Management and Information System Research Group, National School of Business & Management, Abdelmalek Essaadi University, Tangier, Morocco

**Keywords:** Structural equation modeling, Micro-business, Islamic financing

## Abstract

Although microenterprises constitute a significant part of Morocco's economy, they encounter financing difficulties which represent the biggest obstacles to their growth, therefore, Islamic financial institutions can take a prominent role in fostering financial inclusion in Morocco. Accordingly, this article aims to explore the determinants of intention to adopt Islamic financing in micro-business. For this purpose, this study utilizes data collected from a selected sample of Moroccan micro-business owner-managers using face-to-face interviews during March 2021. The results from the partial least squares analysis indicate that religious obligation and Islamic financing reputation positively affect attitude towards Islamic financing. Findings also reveal the positive and significant influence of subjective norms, attitude towards Islamic financing, and perceived behavioral control on intention to adopt Islamic financing.

• This method article can be used to explore the antecedent of Islamic financing adoption in micro‐business.
• Access to this method article may help Islamic bank managers to set up marketing policies in order to strengthen the micro-business owners' intention to adopt Islamic financing.

## Graphical abstract

**Figure:**
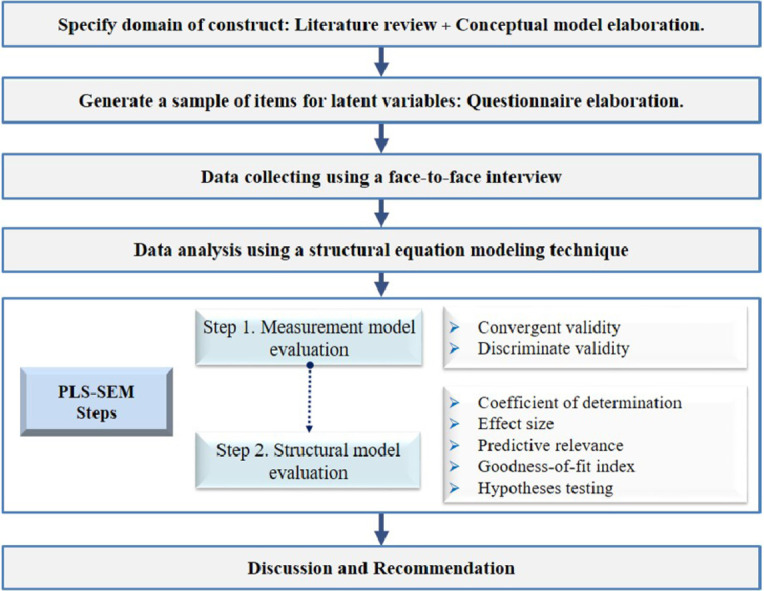

Specifications Table

**Table:** 

Subject Area:	Economics and Finance
More specific subject area:	Finance and Banking
Method name:	Method of Partial least squares path modeling using SmartPLS
Name and reference of original method:	Partial least squares structural equation modeling [1]
Resource availability:	The research dataset is available on a public repository: Repository name: Mendeley Data Data identification number (DOI): 10.17632/r73fyhg3t4.3 Direct URL to data: https://data.mendeley.com/datasets/r73fyhg3t4/3

## Introduction

Islamic financing (IF) is introduced as an alternative to conventional financing to satisfy Muslims' particular requirements by offering them a financial service in accordance with the Islamic religious law (Syariah) [2], based on the prohibition of uncertainty (Gharar), interest (Riba), and gambling (Maysir) [3]. Thereby, since it was lanced in 2017 by Bank Al-Maghrib and under the control of Morocco's Supreme Council of Ulemas, Islamic banks (namely, participatory banks) have emerged as a promising alternative that can strengthen the financial and economic inclusion of microbusiness and attract a new category of investors.

Nowadays, there are five Islamic banks (namely, Umnia Bank, Assafa Bank, BTI Bank, Al Akhdar Bank, and Bank Al Yousr), and three conventional banks, including BMCI Najmah, Arreda of *Credit du Maroc*, and Dar Al-Amane of *Société Générale*. These banks have experienced significant growth, recording an increase of their branch network from 100 in 2018 to 154 branches in 2020. To sustain this growth, Islamic banks need to attract new customers, especially micro-business owners, therefore, it becomes crucial to identify the factors influencing micro-business managers' intention to adopt Islamic financing in business.

The existing literature has investigated the determinants of Islamic financing adoption in different countries, i.e.: Malaysia [4,5], Pakistan [6,7], Uganda [8,9], Tunisia [10], and Ghana [11]. However, the identification of factors that enhance IF among Moroccan micro-business has not been addressed. Therefore, this study aims to address the following research question: Which factors may attract microbusiness owners to adopt Islamic financing?

The remainder of this method article is arranged as follows. Section 2 outlines a literature review regarding factors influencing Islamic financing adoption. Then, Section 3 describes the adopted methodology approach. Section 4 reports and discusses the study's findings. Finally, Section 5 provides conclusions by indicating a number of recommendations.

## Literature review

The study of the determinants of Islamic financing adoption has been subject to considerable scientific papers [5], [6], [7],^9^,[12], [13], [14], which frequently mobilized the theory of reasoned action (TRA) [15], and the theory of planned behavior (TPB) [16]. As an extension of the TRA, the TPB emphasizes that attitude, subjective norms, and perceived behavioral control positively affect individual intention, which turns to enhance the individual's behavior [17].

Based on the TRA, Amin et al. [4] argued that social influence, attitude, and pricing positively affect intention to use IF. In addition, Aziz and Afaq [6] found that attitude and subjective norms positively affect intention to adopt IB in Pakistan, also, they identified uncertainty, awareness, compatibility, and relative advantage as determinants of attitude. Likewise, Mahdzan et al. [14] argued that the understanding of IB concepts and perceived advantage positively affect the adoption of IB services. Bananuka et al. [8] empirically confirmed that attitude and religiosity influence positively intention to adopt IB among micro-business managers in a non-Islamic country. As well, attitude acts as a mediating variable in the association between religiosity and intent to adopt IB [18]. Furthermore, Kaabachi and Obeid [10] found that relative advantage, bank reputation, perceived risk, compatibility, and perceived complexity contribute to the explication of the behavioral intention to adopt IB Services. More recently, Mbawuni and Nimako [11] have empirically verified that attitude, knowledge, perceived innovation, willingness to comply with Islamic law, and perceived benefits are the most significant drivers of intention to adopt IB.

**Fig. 1:**
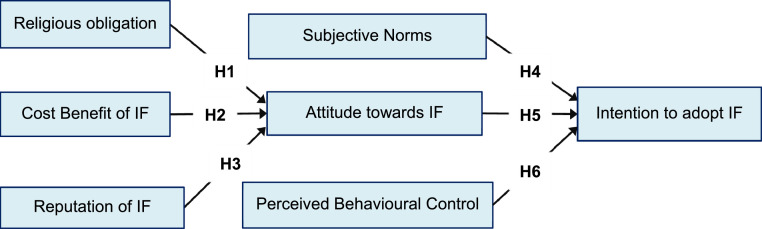
Research model.

In the Halal industry, Jaffar and Musa [13] proposed awareness, religion obligation, cost benefit, business support, and reputation as determinants of attitude towards IF. They further argued that subjective norms, attitude, and behavioral control positively influence on intention to adopt IF in business.The relationships between these variables are also confirmed later by Jaffar and Musa [5]. Accordingly, we assume the following hypotheses (Fig. 1).

H1 Religious obligation has a positive impact on attitude towards IF in business.
H2 Cost benefit of IF has a positive impact on attitude towards IF in business.
H3 Reputation of IF has a positive impact on attitude towards IF in business.
H4 Subjective norm has a positive impact on intention to adopt IF in business.
H5 Attitude towards IF has a positive impact on intention to adopt IF in business.
H6 Perceived behavioral control has a positive impact on intention to adopt IF in business.

## Method details

**Fig. 2:**
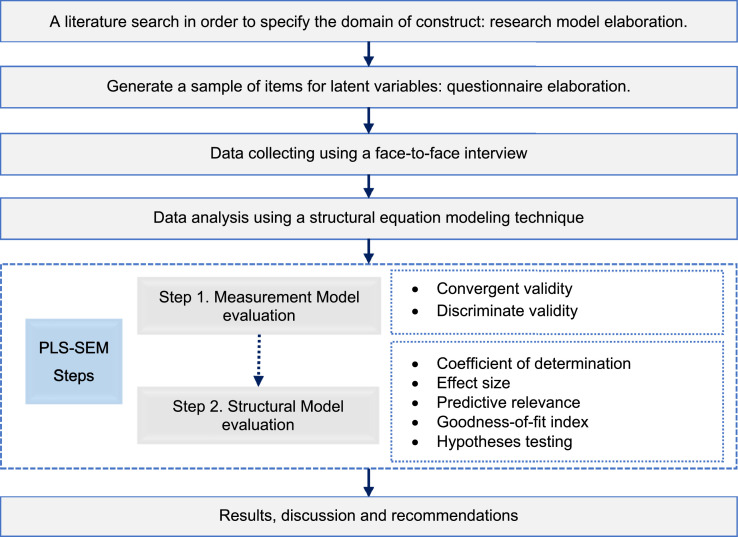
Research steps.

In order to identify the determinants of the intention to adopt Islamic financing among Moroccan micro-business owner-managers, we have opted for a quantitative approach using structural equation modeling under the PLS approach (Fig. 2).

### Research questionnaire

The questionnaire items used in this study were drawn from previous empirical studies [13]. Data of this study include eight latent variables, namely religion obligation (5 items), cost benefits (4 items), the reputation of Islamic financing (3 items), attitude towards Islamic financing (5 items), subjective norms (4 items), perceived behavioral control (3 items), and intention to adopt Islamic financing (3 items). To measure each variable, we adopted the seven-point Likert-type scale. The agreement options are from one (1 = Strongly Disagree) to seven (7 = Strongly Agree).

### Data collection and sampling

The questionnaire was conducted in two stages. In the first stage, a pre-testing phase of the questionnaire items via face-to-face interviews among Moroccan micro-business owner-managers was conducted to ensure the questionnaire content validity. The adjusted questionnaire was translated into Arabic to facilitate participants' understanding of the questions, and thus, it is structured in two sections, the first one focuses on collecting data regarding the socio-demographic profile (age gender, marital status, educational level), and business characteristics (nature of business, age of the enterprise, number of employees), while the second section measures the seven latent variables of the current study (religious obligation, cost benefits, reputation, attitude towards Islamic financing, subjective norms, perceived behavioral control, and intention to adopt Islamic financing).

**Fig. 3:**
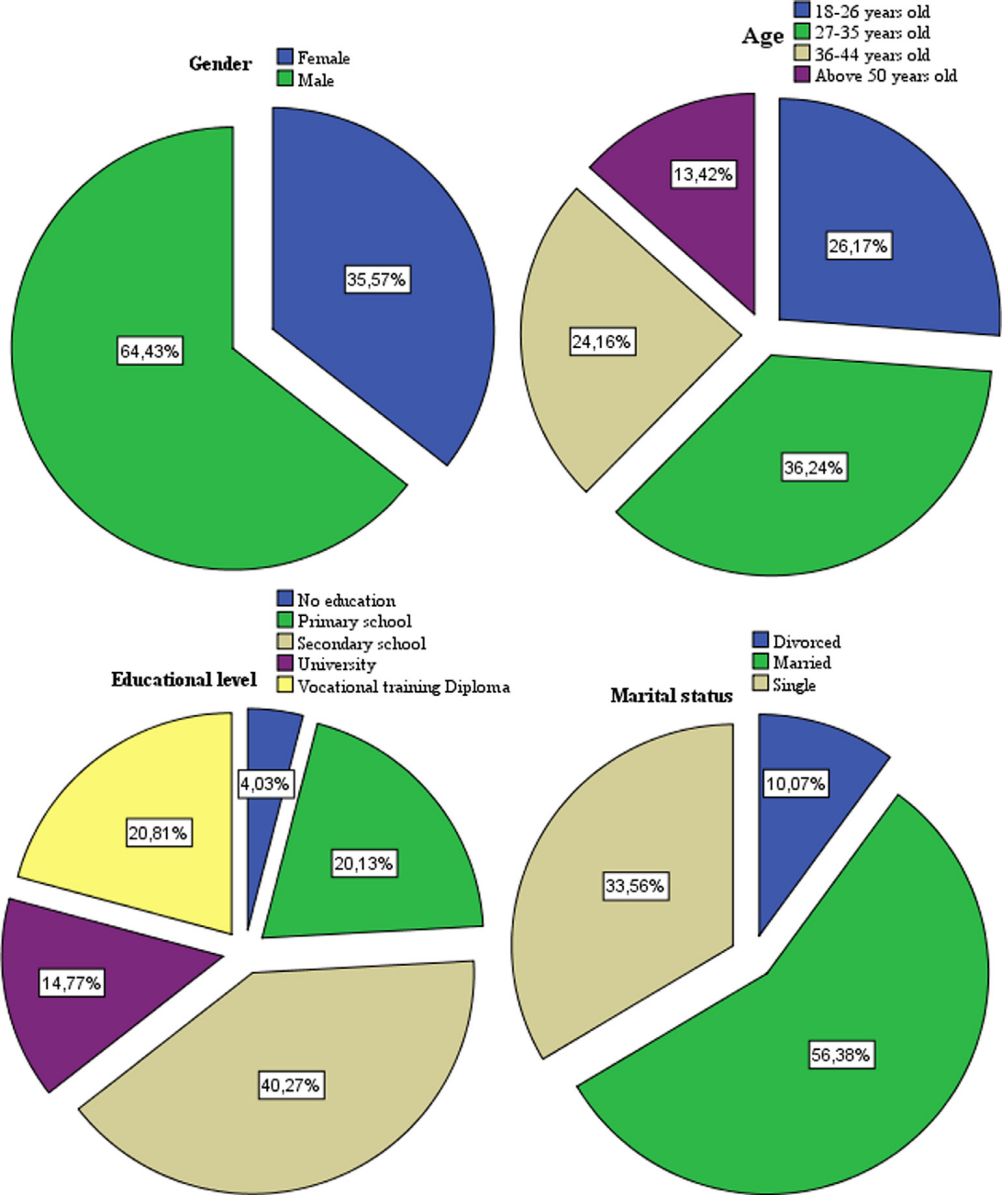
Demographic profile of the survey respondents.

**Fig. 4:**
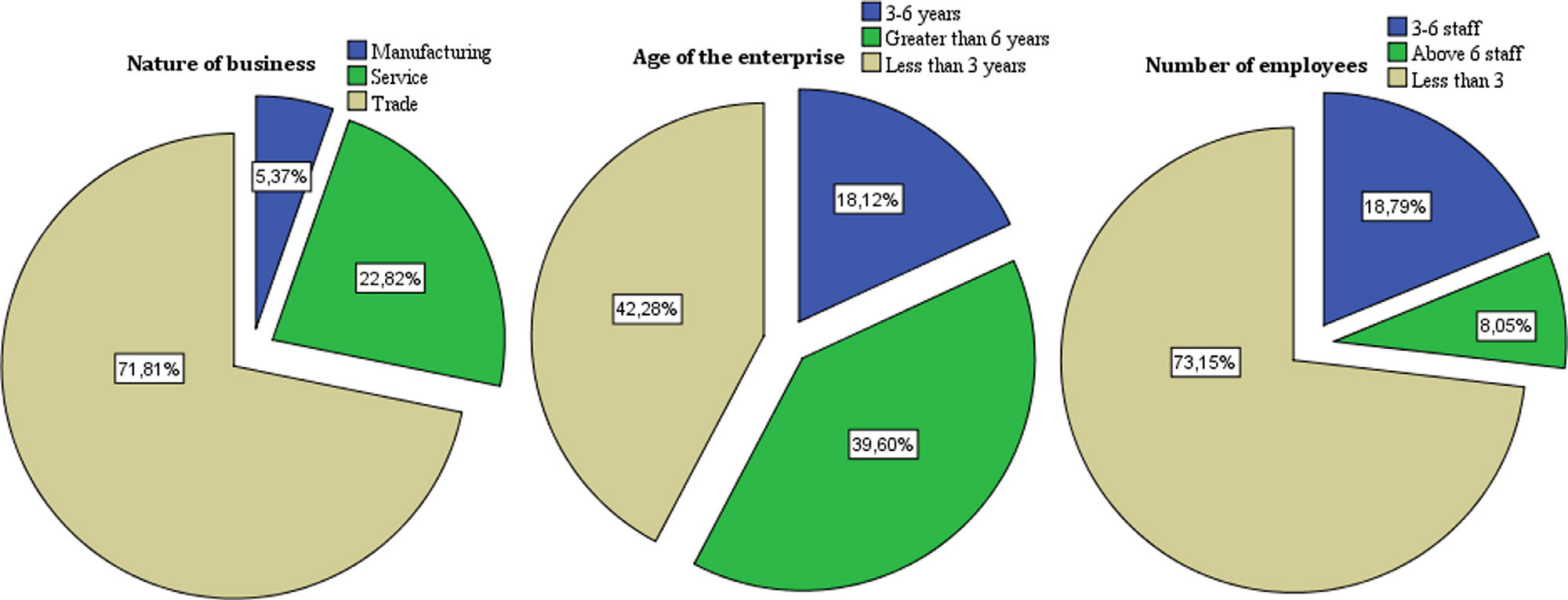
Business characteristics.

In the second stage, four students trained participated in data collection using face-to-face interviews among micro-business owner-managers throughout March 2021. During this period, a total of 149 usable responses were collected from 96 men (64.43%) and 53 women (35.57%), belonging to the age group of 27–35 years (36.2%), followed by the age group of 18–26 years (26.2%), and the age group of 36–44 years (24.2%). 56.4% of surveyed are married, 33.6% are single, and 10.1% are divorced. 40.3% of them have a secondary education level, 20.8% hold a vocational training diploma, and 14.8% studied in university (Fig. 3). As shown in Fig. 4, the majority of respondents were owner-managers of commercial microbusiness (71.81%), with fewer than three employees (73.15%), where 42.28% have been in activity for fewer than three years.

**Fig. 5:**
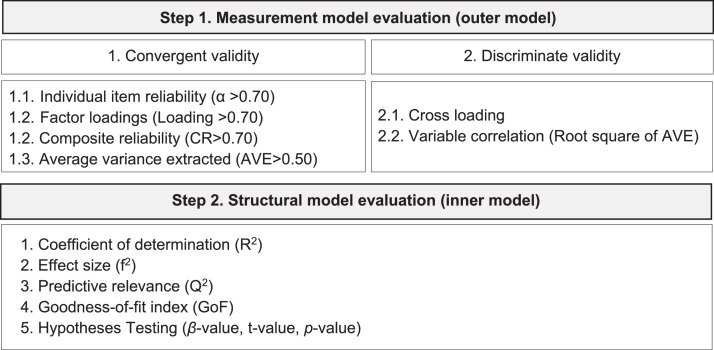
Data analysis - using PLS-SEM & SmartPLS.

For data analysis, we have used the partial least squares structural equation modeling approach [19,20], because this technique provides high levels of statistical power with small sample sizes [21]. Practically the implementation of PLS-SEM through SmartPLS software implies evaluating the measurement model and the structural model (Fig. 5). First, the measurement model evaluation is carried out by assessing convergent validity and discriminant validity. Then, the inner model evaluation requires checking a number of criteria including the coefficient of determination, the effect size, the predictive relevance, the goodness of fit of the model, and the hypothesis testing [20].

## Results and discussions

**Fig. 6:**
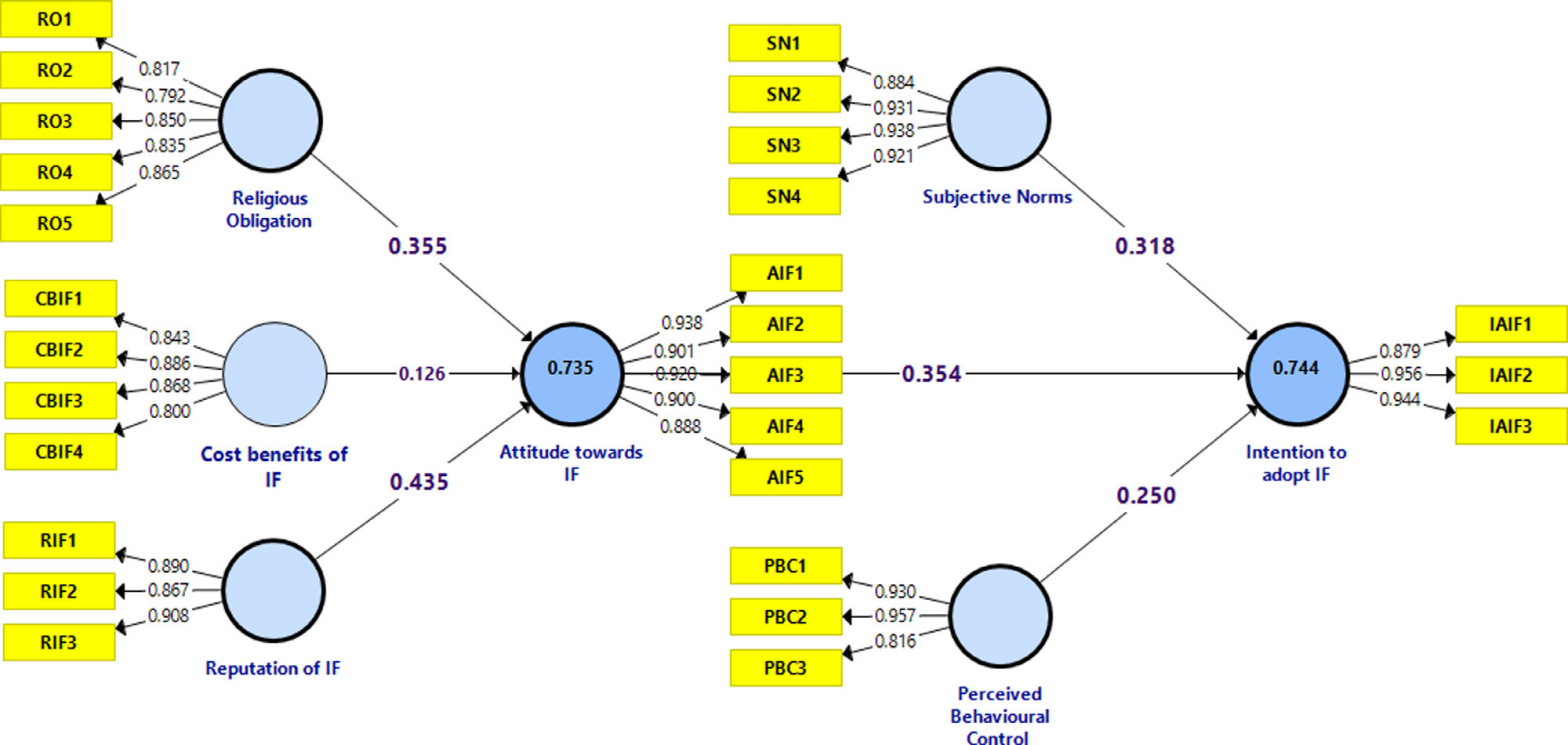
Results of outer model assessment - output SmartPLS.

The measurement model assessment shows a solid convergent validity, according to several criteria: loading, Cronbach's alpha, composite reliability (CR), and average variance extracted (AVE). Thereby, the Cronbach's alpha and composite reliability values are above 0.7. The values of AVE are all above 0.5 (Table 1). Likewise, the values of indicators’ outer loading are above 0.792 (Fig. 6), which confirms their significant contribution to model constructs.

**Table 1:** Convergent validity: Loading, Cronbach's Alpha, rho_A, CR, and AVE.

Constructs	Items	Loading > 0.7	α > 0.7	rho_A > 0.7	CR > 0.7	AVE > 0.5
**Religious obligation**	RO1	0.817	0.889	0.898	0.919	0.693
	RO2	0.792				
	RO3	0.850				
	RO4	0.835				
	RO5	0.865				
**Cost benefits of Islamic financing**	CBIF1	0.843	0.871	0.878	0.912	0.722
	CBIF2	0.886				
	CBIF3	0.868				
	CBIF4	0.800				
**Reputation of Islamic financing**	RIF1	0.890	0.867	0.869	0.918	0.789
	RIF2	0.867				
	RIF3	0.908				
**Attitude towards Islamic financing**	AIF1	0.938	0.948	0.948	0.960	0.827
	AIF2	0.901				
	AIF3	0.920				
	AIF4	0.900				
	AIF5	0.888				
**Subjective norm**	SN1	0.884	0.938	0.940	0.956	0.844
	SN2	0.931				
	SN3	0.938				
	SN4	0.921				
**Perceived behavioral control**	PBC1	0.930	0.886	0.914	0.929	0.815
	PBC2	0.957				
	PBC3	0.816				
**Intention to adopt Islamic financing**	IAIF1	0.879	0.917	0.923	0.948	0.859
	IAIF2	0.956				
	IAIF3	0.944

The PLS outputs illustrated in the Table 2 allow us to assess discriminant validity based on Fornell and Larcker criteria. As shown in Table 3, the loadings of the indicators are greater than all its cross-loadings, supporting the discriminant validity of the outer models.

**Table 2:** Fornell and Larcker criterion.

Construct	AIF	CBIF	IAIF	PBC	RO	RIF	SN
**AIF**	**0.910**						
**CBIF**	0.751	**0.850**					
**IAIF**	0.829	0.757	**0.927**				
**PBC**	0.796	0.706	0.770	**0.903**			
**RO**	0.801	0.777	0.731	0.720	**0.832**		
**RIF**	0.820	0.804	0.799	0.702	0.800	**0.888**	
**SN**	0.868	0.769	0.813	0.752	0.717	0.770	**0.919**

**Table 3:** Results of discriminant validity - loading and cross-loading criteria.

	**AIF**	**CBIF**	**IAIF**	**PBC**	**RO**	**RIF**	**SN**
**AIF1**	**0.938**	0.716	0.781	0.725	0.739	0.764	0.808
**AIF2**	**0.901**	0.650	0.729	0.713	0.773	0.756	0.735
**AIF3**	**0.920**	0.732	0.726	0.718	0.759	0.773	0.816
**AIF4**	**0.900**	0.638	0.767	0.708	0.683	0.705	0.805
**AIF5**	**0.888**	0.678	0.766	0.755	0.687	0.730	0.783
**CBIF1**	0.620	**0.843**	0.588	0.558	0.649	0.676	0.615
**CBIF2**	0.722	**0.886**	0.726	0.656	0.729	0.749	0.725
**CBIF3**	0.575	**0.868**	0.606	0.571	0.628	0.649	0.634
**CBIF4**	0.620	**0.800**	0.639	0.605	0.622	0.647	0.629
**IAIF1**	0.716	0.689	**0.879**	0.672	0.681	0.688	0.686
**IAIF2**	0.775	0.700	**0.956**	0.713	0.659	0.761	0.780
**IAIF3**	0.810	0.718	**0.944**	0.754	0.694	0.767	0.790
**PBC1**	0.792	0.656	0.761	**0.930**	0.686	0.677	0.729
**PBC2**	0.746	0.674	0.748	**0.957**	0.697	0.690	0.698
**PBC3**	0.598	0.579	0.552	**0.816**	0.552	0.515	0.602
**RO1**	0.602	0.592	0.579	0.574	**0.817**	0.595	0.525
**RO2**	0.584	0.589	0.537	0.594	**0.792**	0.577	0.520
**RO3**	0.711	0.671	0.634	0.659	**0.850**	0.751	0.643
**RO4**	0.631	0.675	0.583	0.481	**0.835**	0.699	0.596
**RO5**	0.774	0.695	0.688	0.670	**0.865**	0.693	0.675
**RIF1**	0.754	0.745	0.726	0.629	0.710	**0.890**	0.752
**RIF2**	0.681	0.686	0.702	0.632	0.689	**0.867**	0.660
**RIF3**	0.747	0.711	0.701	0.613	0.733	**0.908**	0.639
**SN1**	0.711	0.670	0.715	0.677	0.632	0.661	**0.884**
**SN2**	0.785	0.669	0.731	0.700	0.623	0.693	**0.931**
**SN3**	0.813	0.729	0.752	0.678	0.662	0.713	**0.938**
**SN4**	0.872	0.754	0.786	0.707	0.714	0.758	**0.921**

The structural model assessment requires the verification of the coefficient of determination, the effect size, the predictive relevance, and the Goodness-of-Fit index. The R-square values for the two endogenous latent variables, attitude towards Islamic financing, and intention to adopt Islamic financing are above 0.67, which are 0.735 and 0.744, respectively. This shows a substantial level of determination of these variables (Table 4).

**Table 4:** Coefficient of determination of the endogenous constructs**.**

Endogenous constructs	R Square	R Square adjusted
Attitude towards IF	0.735	0.730
Intention to adopt IF	0.744	0.739

As shown in Table 5, the effect size value of reputation on attitude towards Islamic financing is 0.197, which is considered as a medium effect size. The religious obligation has a small effect size on attitude towards Islamic financing (0.02 ≤ f^2^ < 0.15). The same for the effect size of the attitude towards IF, the perceived behavioral control, the perceived usefulness of IF and subjective norms on intention to adopt IF. Further, Table 6 shows that the predictive relevance values (Q Square) are greater than zero, which support the predictive relevance of the model. Likewise, the goodness-of-fit value (GoF = 0.76) is large enough to support the overall validity of the PLS model (Table 7).

**Table 5:** Effect size**.**

Constructs			f^2^ values	Signification
Religious obligation	→	Attitude towards IF	0.147	Small effect size
Cost benefits of IF	→	Attitude towards IF	0.018	No effect size
Reputation of IF	→	Attitude towards IF	0.197	Medium effect size
Attitude towards IF	→	Intention to adopt IF	0.098	Small effect size
Perceived Behavioral Control	→	Intention to adopt IF	0.086	Small effect size
Subjective Norms	→	Intention to adopt IF	0.094	Small effect size

**Table 6:** Predictive relevance - Q square.

Constructs	SSO	SSE	Q² (=1 − SSE/SSO)
**Attitude towards IF**	745.000	296.423	**0.602**
Cost benefits of IF	596.000	596.000	
**Intention to adopt IF**	447.000	165.052	**0.631**
Perceived Behavioral Control	447.000	447.000	
Religious Obligation	745.000	745.000	
Reputation of IF	447.000	447.000	
Subjective Norms	596.000	596.000

**Table 7:** Goodness of fit of the model calculation.

Constructs	R Square	AVE	Goodness of Fit (GoF)
Attitude towards IF	0.735	0.827	$\sqrt{\bar{\;R^{2}}\times \bar{\text{AVE}}}$
Intention to adopt IF	0.744	0.859	
Religious Obligation		0.693	
Cost benefits of IF		0.722	$\sqrt{0.7395\;\times \;0.7927}$
Reputation of IF		0.789	
Subjective Norms		0.844	
Perceived Behavioral Control		0.815	**GoF** = 0.76564

**Fig. 7:**
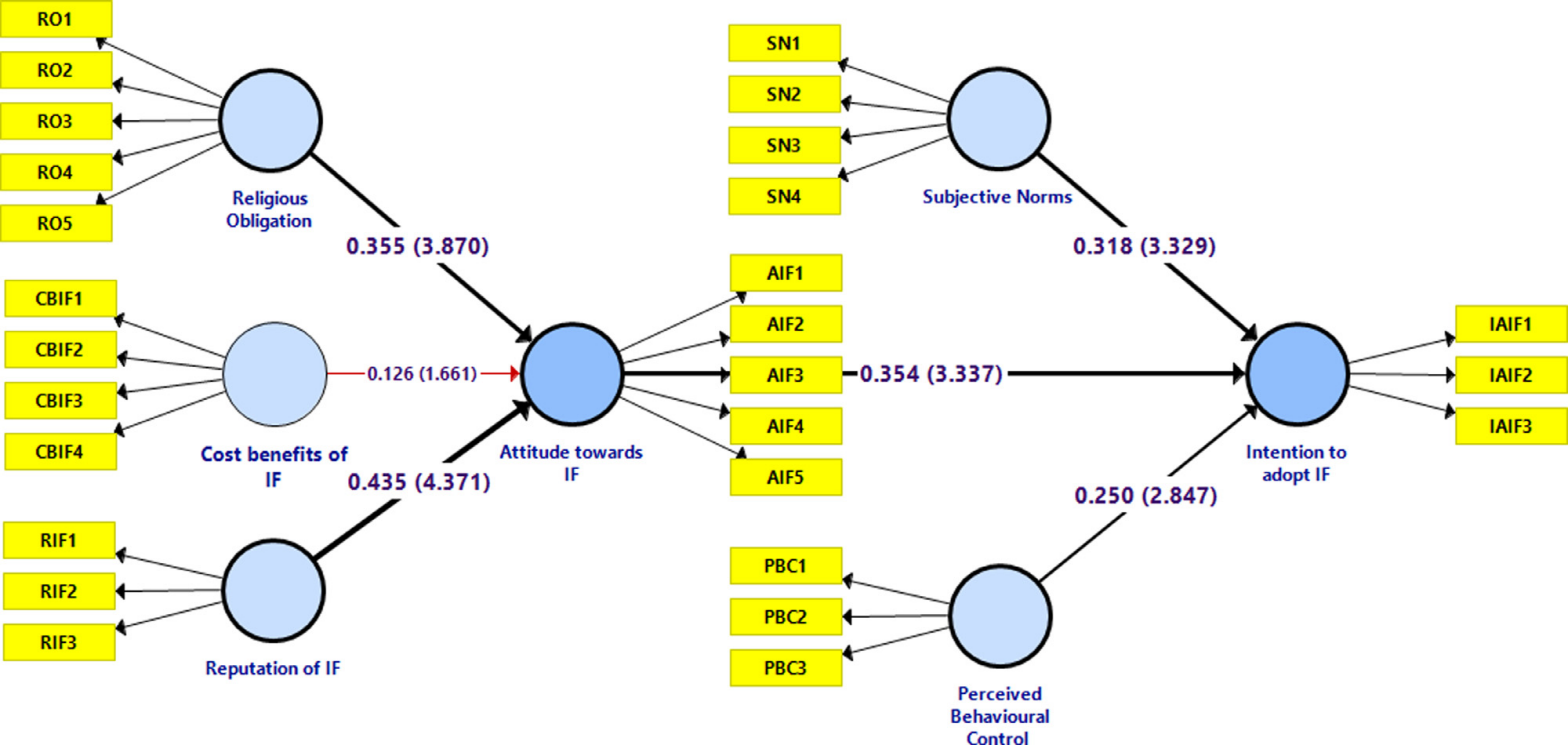
Evaluated model.

Fig. 7 summarizes the results of hypotheses testing, which reveal that attitude towards Islamic financing depends upon two variables, i.e., religious obligation and reputation of Islamic financing. Additionally, these findings suggest that attitude towards IF, perceived behavioral control, and subjective norms contribute positively and significantly to the explanation of the intention to adopt Islamic financing among micro-business owners.

**Fig. 8:**
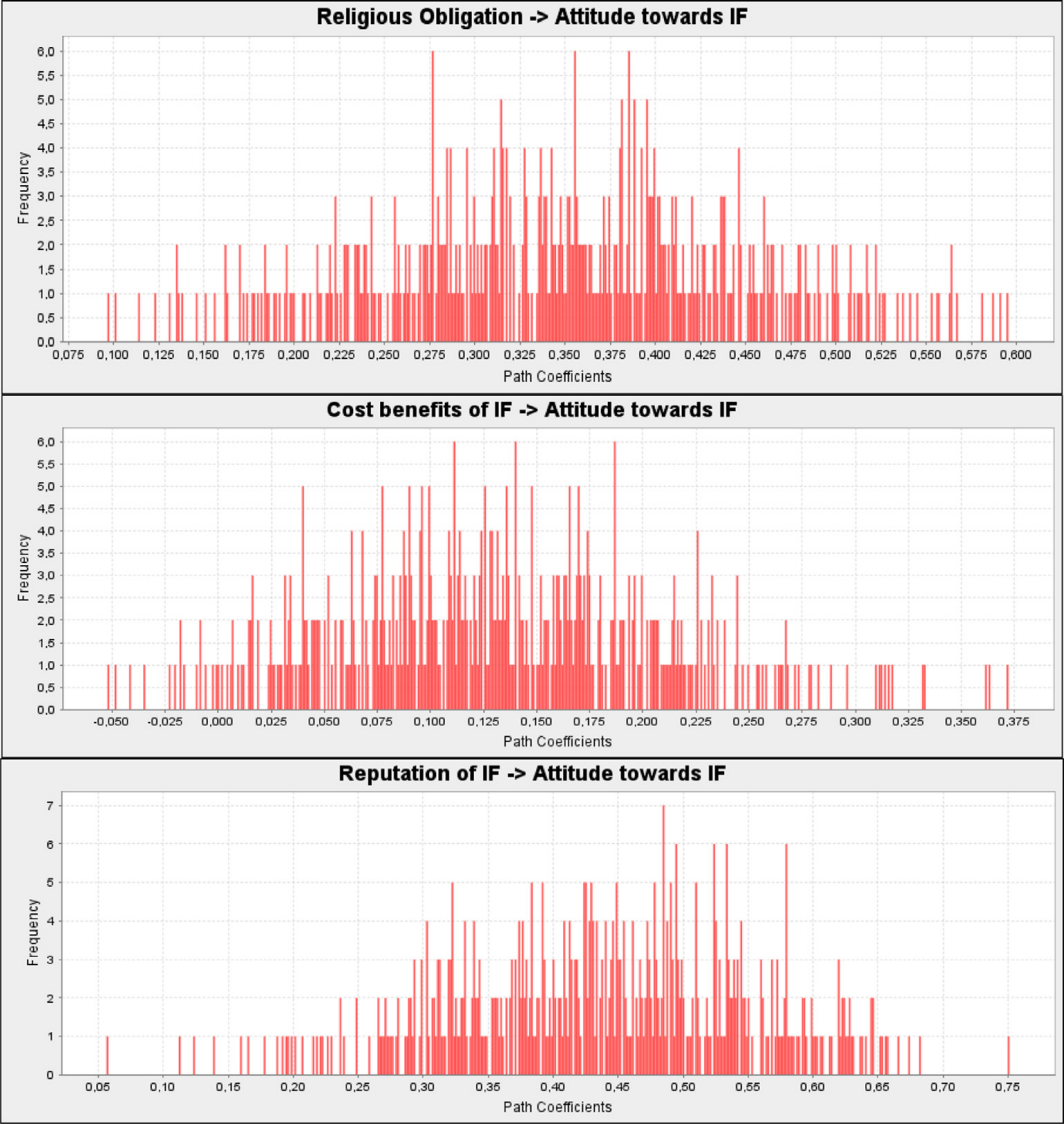
Path coefficients histogram - attitude towards IF determinants.

From the findings (Fig. 8), it turned out that religious obligation (H1. *β* = 0.335, *t* = 3.870, *p* < 0.00), and reputation of IF (H3. *β* = 0.435, *t* = 4.371, *p* < 0.00) has a positive and significant effect on attitude towards IF. In contrary, cost benefits of IF (H2. *β* = 0.126, *t* = 1.661, *p* = 0.097) has no significant effect on attitude towards IF.

**Fig. 9:**
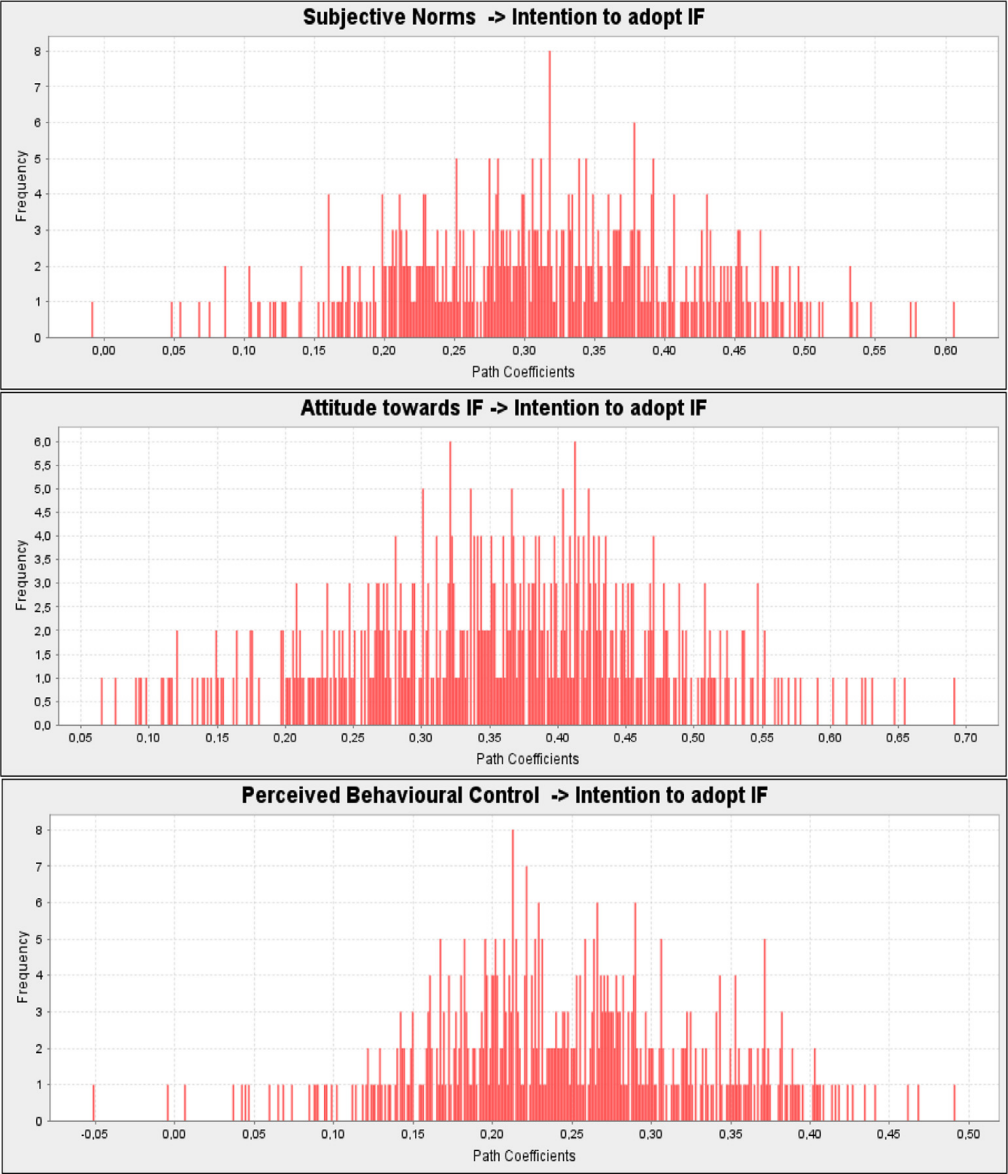
Path coefficients histogram- Intention to adopt IF determinants.

Based on the results of the PLS-SEM analyzes (Fig. 9), all hypotheses relationships that assume a positive relationship between subjective norms (H4. *β* = 0.318, *t* = 3.329, *p* = 0.001), attitude towards IF (H5. *β* = 0.354, *t* = 3.337, *p* = 0.001), perceived behavioral control (H6. *β* = 0.250, *t* = 2.847, *p* = 0.005), and intention to adopt IF were supported.

The study findings reveal that religious obligation and reputation of Islamic financing positively affect attitude towards Islamic financing. These results are consistent with the previous studies [5,13], which suggest that Islamic financing reputation and religious obligation are determinants of intention to adopt IB among entrepreneurs of halal micro and SMEs. The PLS-SEM analysis also confirms the direct and positive association between attitude towards IF, perceived behavioral control, subjective norms, and intention to adopt IF in business. These findings are in line with prior studies [5,^6^,13], which conclude that attitude, behavioral control, and subjective norms significantly and directly influence on intention to adopt IF.

## Conclusion

The current study aimed to identify factors that contribute to enhancement of intention to adopt Islamic financing in business. Relying on the partial least square path modeling, the findings emphasize that both religious obligation and Islamic financing reputation provide a foundation to enhance attitude towards Islamic financing. Further, attitude towards IF, subjective norms, and perceived behavioral control have been identified as significant determinants of intention to adopt IF in micro-business. Thus, our findings provide insights into how Islamic bank managers could establish marketing policies in order to attract micro-business owners’, by enhancing their level of intention to adopt IF. At this level, Islamic banks marketing managers need to build up a favourable attitude regarding IF by informing potential customers about Islamic banking products/services, and undertaking more efforts to spread Islamic financial knowledge through raising awareness about the advantages of IF among Moroccan micro-business owners.

## Declaration of Competing Interest

The authors declare that they have no known competing financial interests or personal relationships that could have appeared to influence the work reported in this paper.
